# The impact of COVID-19 pandemic on breast surgery in Italy: a multi-centric retrospective observational study

**DOI:** 10.1007/s13304-023-01474-y

**Published:** 2023-03-06

**Authors:** R. Sgarzani, G. Macrì, A. Gurrado, A. Curcio, F. De Lorenzi, V. Galimberti, C. Garusi, M. Bocchiotti, M. Roncella, F. Rovera, G. Caputo, A. Sgarella, L. Barone Adesi, D. Terribile, R. Nonnis, P. Frittelli, B. Cagli, S. Tenna, I. Baldelli, A. Cordova, R. Elia, M. Salgarello

**Affiliations:** 1 Dipartimento DIMEC, Università Di Bologna, Via Massarenti 9, 40126 Bologna, Italy; 2 U.O. Chirurgia Plastica, Policlinico Universitario Agostino Gemelli, Rome, Italy; 3 Unit of Academic General Surgery “V. Bonomo”, Department of Biomedical Sciences and Human Oncology, University of Bari, “A. Moro”, Bari, Italy; 4 Chirurgia Senologica, AUSL Della Romagna, Forlì, Italy; 5 Department of Plastic Surgery, European Institute of Oncology, Milan, Italy; 6 U.O. Chirurgia Plastica, Città Della Salute, Turin, Italy; 7 UOC Chirurgia Senologica, Azienda Ospedaliera Universitaria Pisana, Pisa, Italy; 8 ASST SetteLaghi Varese, Università Degli Studi Dell’Insubria, Varese, Italy; 9 U.O. Chirurgia Plastica, ASUFC, Udine, Italy; 10 U.O. Chirurgia Generale 1, IRCCS- Policlinico San Matteo, Pavia, Italy; 11 Chirurgia Plastica, AOU Sassari, Sassari, Italy; 12 U.O. Chirurgia Senologica, Ospedale San Giovanni Calibita Fatebenefratelli Isola Tiberina, Rome, Italy; 13 U.O. Chirurgia Plastica, Campus Bio-Medico, Rome, Italy; 14 U.O. Chirurgia Plastica, Ospedale San Martino, Genoa, Italy; 15 U.O. Chirurgia Plastica, Azienda Ospedaliera Universitaria Paolo Giaccone, Palermo, Italy; 16 Unit of Academic Plastic Surgery, Department of Emergency and Organ Transplantation, University of Bari, “A. Moro”, Bari, Italy

**Keywords:** COVID-19, Pandemic, Breast surgery, Mastestomy, DTI reconstruction

## Abstract

COVID-19 pandemic had an impact on surgical activities. The aim of this multi-centric, retrospective study was to evaluate the impact of the COVID-19 pandemic on breast surgery. The patients who operated during the pre-pandemic year 2019 were compared to those operated in 2020. Fourteen Breast Care Units provided data on breast surgical procedures performed in 2020 and 2019: total number of breast-conserving surgery (BCS), number of 1st level oncoplastic breast surgery (OBS), number of 2nd level OBS; total number of mastectomies, mastectomies without reconstruction, mastectomies with a tissue expander, mastectomies with direct to implant (DTI) reconstruction, mastectomies with immediate flap reconstruction; total number of delayed reconstructions, number of expanders to implant reconstructions, number of delayed flap reconstructions. Overall 20.684 patients were included: 10.850 (52.5%) operated during 2019, and 9.834 (47.5%) during 2020. The overall number of breast oncologic surgical procedures in all centers in 2020 was 8.509, compared to 9.383 in 2019 (− 9%). BCS decreased by 744 cases (− 13%), the overall number of mastectomies decreased by 130 cases (− 3.5%); mastectomy-BCS ratio was 39–61% in 2019, and 42–58% in 2020. Regarding immediate reconstructive procedures mastectomies with DTI reconstruction increased by 166 cases (+ 15%) and mastectomies with immediate expander reconstruction decreased by 297 cases (− 20%). Breast-delayed reconstructive procedures in all centers in 2020 were 142 less than in 2019 (− 10%). The outburst of the COVID-19 pandemic in 2020 determined an implemented number of mastectomies compared to BCS, an implemented number of immediate breast reconstructions, mainly DTI, and a reduction of expander reconstruction.

## Introduction

2020 is a year that none of us will forget; the outburst of the COVID-19 pandemic in absence of an effective vaccine, until the end of 2020 [1], challenged the Health care systems. Italy was the first country in Europe hit by the emergency [2]: on January 31st 2020, the Italian Government declared a 6 months national emergency, when two Chinese tourists from Wuhan, positive for SARS-CoV-2, were detected in Rome. The first Italian patient was a 38-year-old man, detected on February 20th. Italy was the first to introduce stringent social containment by closing schools and universities on March 4th and then establishing a total lockdown on 22nd March. In the beginning, Northern Regions were the most involved, especially Lombardy with a peak of more than 23.000 excess deaths 2 months after the beginning of the first wave (+ 118% compared to the average mortality rate) [2].

Italian Health care system had to face workforce, facilities, and medical device shortages.

Hospitals had to reduce surgical procedures to allow the use of ventilators, hospital space and personnel for COVID-19 suffers, and patients could not undergo regular check-ups, like breast screening [3], or were even afraid to go to the hospital for scheduled surgery [4]. During 2020 the surgical guidelines evolved continuously with real-time experience, depending on the peak of the pandemic.

The study’s aim was to evaluate the impact of the COVID-19 pandemic on the surgical activity of the Italian Breast Care Units, comparing the breast surgical procedures performed in the pre-pandemic year 2019 to those performed in 2020.

## Materials and methods

This is a multi-centric retrospective observational study on data collected from 14 Breast Care Units in different countries of Italy, all affiliated to the Beautiful After Breast Cancer Italia Onlus (www.beautifulabc.it) project “Women for Women” for Breast Reconstruction Awareness (BRA) Day 2021. Only Breast Care Units from Italian hospitals that were not converted into Covid Hospitals during the pandemic were involved.

The inclusion criteria were: patients who underwent breast surgical procedures for cancer in 2020, defined as the case group, and during 2019, defined as the control group.

The endpoints of the study were:

- To quantify the overall reduction of surgical procedures for breast cancer;
- To evaluate whether the mastectomy-breast conserving surgery (BCS) ratio was adequately maintained;
- To compare immediate reconstructive procedures (direct-to-implant DTI, immediate flap reconstruction, expander);
- To quantify the overall reduction of breast-delayed reconstructive procedures.

No ethical approval from the institutional board was needed for this study because only aggregate data were collected from each center.

Data collected for each Breast Care Unit:

- Total number of patients operated for breast cancer;
- Total number of breast-conserving surgery (BCS) procedures;
- Total number of 1^st^-level oncoplastic breast surgery (OBS) procedures, and of 2^nd^-level OBS procedures;
- Total number of mastectomies: without reconstruction, with a tissue expander, with DTI, with immediate flap reconstruction;
- Total number of delayed reconstructions, of expander to implant reconstructions, and of delayed flap ones.

**Fig. 1:**
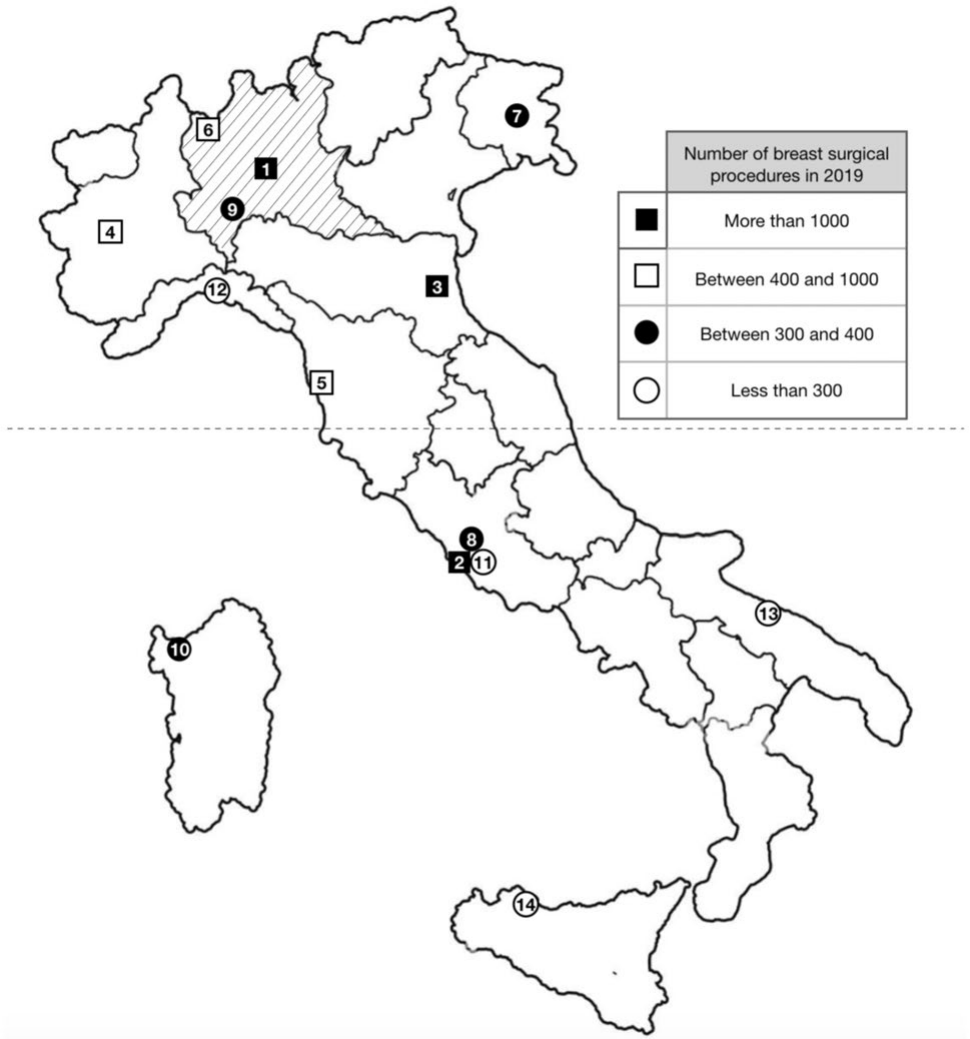
Map of Italy showing the location of the 14 Breast Care Units, classified in the table and numbered according to the number of breast surgical procedures performed in the pre-pandemic year 2019: 1 IEO-Milano, 2 Policlinico Gemelli- Roma, 3 AUSL della Romagna, 4 Città della Salute-Torino, 5 Pisa, 6 ASST Sette Laghi, Varese, 7 ASUFC, Udine, 8 Campus Biomedico, Roma, 9 IRCCS- Policlinico San Matteo- Pavia, 10 Ospedale Sassari, 11 Ospedale Fatebenefratelli- Roma, 12 Ospedale San Martino—Genova, 13 Policlinico Bari, 14 Policlinico Palermo. Collected data are analyzed dividing the 14 Breast Care Units into 3 subgroups according to the geographic location: Lombardy (in stripes on the map); Northern Italy except Lombardy (above the dashed line); Central and Southern Italy (below the dashed line)

Collected data were analyzed by dividing the 14 Breast Care Units into 3 subgroups according to geographic location, to underline how regions hitten differently by the first wave of the pandemic coped with breast surgery:

- Lombardy (in stripes on Fig. 1);
- Northern Italy except Lombardy (above the dashed line in Fig. 1);
- Central and Southern Italy (below the dashed line in Fig. 1).

### Statistical analysis

Statistical analysis was performed with Stata 17 (StataCorp LP, TX); categorical variables were reported as absolute and relative frequencies and compared using the Chi-squared test. The expected frequencies were considered those of 2019 (pre-pandemic year). Results were considered statistically significant in the case of a *p* value < 0.05.

## Results

Data were collected from 14 Italian Breast Care Units. In Fig. 1 Breast Care Units are classified according to the overall number of breast surgical procedures performed in the pre-pandemic year 2019 into more than 1000 procedures per year (3 centers), between 400 and 1000 procedures per year (3 centers), between 300 and 400 procedures per year (4 centers), less than 300 procedures per year (4 centers).

We enrolled in the study 20.684 patients who underwent breast surgery: 10.850 (52.5%) operated during the pre-pandemic year 2019, and 9.834 (47.5%) during 2020.

Data on breast oncologic surgical procedures for 2019 and 2020 are reported in Table 1, and the trend is in Table 2. The overall number of breast oncologic surgical procedures in all centers in 2020 was 8.509, it decreased by 9% compared to pre-pandemic year 2019 (9.383 patients) and the difference was statistically significative (*p* < 0.01).

**Table 1:** Breast oncologic surgery procedures in the pandemic year 2020 and in the pre-pandemic year 2019

			2020	2019	*p*
Breast-conserving surgery (*n,* %)	1st level oncoplasty		4243 (85.7%)	4922 (86.5%)	** < 0.01**
	2nd level oncoplasty		703 (14.2%)	768 (13.4%)	ns
	Tot.		4.946 (58%)	5690 (61%)	** < 0.01**
Mastectomy (*n,* %)	without reconstruction		959 (27%)	963 (26%)	** < 0.05**
	With immediate reconstruction	Tissue expander	1192 (33.4%)	1489 (40.3%)	** < 0.01**
		DTI	1271 (35.6%)	1105 (30%)	** < 0.01**
		Flap	141 (4%)	136 (3.7%)	ns
	Tot.		3563 (42%)	3693 (39%)	** < 0.05**
Tot.			8509	9383	** < 0.01**

Data shown as the absolute frequency with a percentage in brackets. Chi-squared test for ordinal/binomial variables

*DTI* mastectomies with direct to implant

Bold emphasized values are statistically significant

**Table 2:** Breast oncologic surgery trend 2020–2019

Trend 2020–2019	Breast conserving surgery			Mastectomy				
	1st level oncoplasty	2nd level oncoplasty	*Tot.*	Without reconstruction	With immediate reconstruction			Tot
					Tissue expander	DTI	Flap	
Tot (*n*, %)	* − 679 (− 13.8%)*	* − 65 (-8.3%)*	* − 744 (− 13%)*	* − 4 (− 0.4%)*	* − 297 (− 20%)*	+ *166 (*+ *13%)*	+ *5 (*+ *3.6%)*	* − 130 (− 3.5%)*

The mastectomy/BCS rate increased significantly: mastectomies were 42% of all breast oncologic surgical procedures in 2020 vs 39% in 2019 (*p* < 0.05); BCS procedures were 58% in 2020 vs 61% in 2019 (*p* < 0.01).

In terms of trend analysis 2019–2020 (Table 2), BCS decreased in number: 1st level OBS minus 679 cases (− 13.8%) and 2nd level OBS minus 65 cases (− 8.3%), for a total of minus 744 cases (− 13%). The overall number of mastectomies also decreased by 130 cases (− 3.5%).

Regarding immediate reconstructive procedures, statistical difference was found between direct-to-implant reconstructions, that increased by 15% (1271 in 2020 vs 1105 in 2019; *p* < 0.01) and expander reconstruction, which decreased by 20% (1192 in 2020 vs 1489 in 2019; *p* < 0.01).

Data on breast oncologic surgical procedures for 2019 and 2020 in the 3 subgroups (Lombardy; Northern Italy except Lombardy; Central and Southern Italy) are reported in Table 3.

**Table 3:** Breast oncologic surgery procedures in the pandemic year 2020 and in the pre-pandemic year 2019 in the 3 subgroups: Lombardy; Northern Italy except Lombardy; Central and Southern Italy

Lombardy			2020	2019	*p*
Breast-conserving surgery	1st level oncoplasty		1406	1866	** < 0.01**
	2nd level oncoplasty		178	142	** < 0.01**
	Tot.		1584 (49%)	2008 (52%)	** < 0.01**
Mastectomy	Without reconstruction		371	372	** < 0.01**
	With immediate reconstruction	Tissue expander	620	822	** < 0.01**
		DTI	591	558	** < 0.01**
		Flap	79	83	ns
	Tot.		1661 (51%)	1835 (48%)	** < 0.05**
Tot.			3245	3843	** < 0.01**
Northern Italy except Lombardy			2020	2019	*p*
Breast-conserving surgery	1st level oncoplasty		1501	1809	< 0.01
	2nd level oncoplasty		276	329	ns
	Tot.		1777 (58%)	2138 (63%)	** < 0.01**
Mastectomy	Without reconstruction		448	460	ns
	With immediate reconstruction	Tissue expander	443	482	ns
		DTI	356	293	** < 0.01**
		Flap	14	18	ns
	Tot.		1261 (42%)	1253 (37%)	** < 0.01**
Tot.			3038	3391	ns
Central and Southern Italy			2020	2019	*p*
Breast-conserving surgery	1st level oncoplasty		1336	1247	ns
	2nd level oncoplasty		249	297	** < 0.01**
	Tot.		1585 (71%)	1544 (72%)	ns
Mastectomy	Without reconstruction		140	131	ns
	With immediate reconstruction	Tissue expander	129	185	< 0.01
		DTI	324	254	** < 0.01**
		Flap	48	35	ns
	Tot.		641 (29%)	605 (28%)	ns
Tot.			2226	2149	** < 0.01**

Data shown as absolute frequency. Chi-squared test for ordinal/binomial variables

*DTI* mastectomies with direct to implant

Bold emphasized values are statistically significant

We can summarize that:

- Lombardy and Northern Italy, but not Central and Southern Italy contributed to the reduction of the overall number of breast oncologic surgical procedures;

- All groups contributed to the overall increase in mastectomy/BCS rate;

- Lombardy and Northern Italy, but not Central and Southern Italy contributed to the overall BCS number decrease;

- Lombardy was the only group with a mastectomy number decrease, that determined the overall mastectomy number decrease;

- All groups presented an increased number of DTI reconstructions;

- All groups presented a reduced number of immediate reconstructions with the expander.

Data on breast-delayed reconstructive procedures for 2019 and 2020 and the trend are reported in Table 4.

**Table 4:** Delayed breast reconstruction procedures in pandemic year 2020 and in pre-pandemic year 2019 and delayed breast reconstruction procedures trend 2020–2019

		**2020**	**2019**	***p***	**Trend** **2020–2019**
**Delayed reconstruction** (*n,* %)	Expander to implant	1225 (92%)	1336 (91%)	*ns*	* − 111 (− 8%)*
	Flap	100 (8%)	*131* (9%)	*ns*	* − 31 (− 24%)*
	Tot	1325	1467	** < *0.01***	* − 142 (− 10%)*

Data shown as the absolute frequency with a percentage in brackets. Chi-squared test for ordinal/binomial variables

Bold emphasized values are statistically significant

Statistical difference was found between the overall number of breast-delayed reconstructive procedures, which decreased by 10% (1325 vs 1467; *p* < 0.01).

Data on breast-delayed reconstructive procedures for 2019 and 2020 in the 3 subgroups (Lombardy; Northern Italy except Lombardy; Central and Southern Italy) are reported in Table 5; all groups contributed to the overall reduction of delayed reconstructive procedures.

**Table 5:** Delayed breast reconstruction procedures in the pandemic year 2020 and in the pre-pandemic year 2019 in the 3 subgroups: Lombardy; Northern Italy except Lombardy; Central and Southern Italy

Lombardy		**2020**	**2019**	***p***
**Delayed** **reconstruction**	Expander to implant	675	681	*ns*
	Flap	20	50	** < *0.01***
	tot	695	731	*ns*
Northern Italy except Lombardy				
**Delayed** **reconstruction**	Expander to implant	337	358	** < *0.01***
	Flap	7	13	*ns*
	tot	344	371	** < *0.01***
Central and Southern Italy				
**Delayed reconstruction**	Expander to implant	213	297	** < *0.01***
	Flap	73	68	*ns*
	tot	286	365	** < *0.01***

Data shown as absolute frequency. Chi-squared test for ordinal/binomial variables

Bold emphasized values are statistically significant

## Discussion

In our multi-centric retrospective observational study, we report an overall decrease of 9% in surgical procedures for breast cancer in 2020.

During the early phases of the pandemic, the diagnosis of breast cancer decreased; this was not a real incidence reduction but was mostly due to patients’ refusal to undergo diagnostic appointments and breast biopsies [4]. Delayed cancer diagnosis could be linked on one hand to the fear to go to the hospital and seek medical attention and on the other hand to the suspension and delay of screening and follow-up programs.

In March 2020, indeed, the American Society of Breast Surgeons and the American College of Radiology released the indication to postpone all breast screening exams (screening mammography, ultrasound, and MRI) and to discontinue routine and non-urgent breast health appointments [5]. In Italy, all screening radiological exams were postponed, while urgent symptomatic patients were visited. The Italian College of Breast Radiologists released 4 recommendations [3]: 1. patients scheduled for in-depth diagnostic breast imaging or needle biopsy should confirm the appointment, 2. patients who have suspicious symptoms of breast cancer should request non-deferrable exams, 3. asymptomatic women performing annual mammographic follow-up after breast cancer could preferably schedule the appointment within 1 year and 3 months from the previous check, 4. screening mammography should be re-scheduled within 3 months from the date.

To allow the use of ventilators, hospital space and personnel for COVID-19, patients elective surgery was cancelled and reduced, and cancer care was deprioritized and delayed. Over time this would clearly have led to a collateral increase in the number of deaths. Therefore, suggestions on risk stratification for breast cancer were developed: based on tumour stage and biology, patients were divided into those for whom surgery was time critical and those for whom surgery could be reasonably deferred for a period, like up to 60 days for early-stage breast cancer [6]. In Italy, some hospitals were converted into COVID centers and surgical procedures were cancelled or referred to other hospitals. To avoid an increase in collateral breast cancer-related deaths some other hospitals were recognized as "hub" centers for breast cancer treatment during the pandemic emergency. In hub centers, breast operations were selected balancing the risk of tumor progression and the risk of exposure to COVID-19, according to the Paragraph Priorities for Breast Disease: Surgical Oncology [7].

As an example, IEO Hospital in Milan (Lombardy) was recognized as “hub” center for breast cancer treatment during the pandemic emergency and reported that from 9th March (start of national lock-down) 340 operations were performed in 4 weeks, + 20% compared to the same 4 weeks in 2019 [8].

All the involved Breast Care Units belonged to Italian hospitals that were not converted into Covid Hospitals during the pandemic, therefore, the overall decrease in surgical procedures for breast cancer (9%) may represent only part of the real reduction of operated patients in Italy, that we are, however, unable to quantify.

During the first wave of the pandemic there was a difference between Lombardy, North and South Italy. North Italy, especially Lombardy, was massively attacked by the outbreak, in fact these areas, but not Central and Southern Italy contributed to the reduction of the overall number of breast oncologic surgical procedures.

In our study mastectomy-BCS rate, which was 39–61% in the pre-pandemic year 2019, became 42–58% in 2020, with a significant increase in mastectomies. In particular, 1st level OBS (those where, according to Clough’s classification [9], removed tissue is less than 20%) decreased by 13.8%. Lombardy and Northern Italy, but not Central and Southern Italy contributed to the overall BCS number decrease, but all geographic groups contributed to the increase of mastectomy/BCS rate.

Unfortunately, these data show the important impact of the pandemic on the diagnostic and therefore therapeutic delay in patients with breast cancer. Delayed and later-stage diagnoses as well as uncertainties about the future and the fear of delays in radiotherapy (mandatory adjuvant treatment after BCS), may be responsible for the increased number of indications of mastectomy.

We report an increased number of mastectomies with DTI reconstruction by 13%; however, mastectomies with immediate expander reconstruction decreased by 297 cases (− 20%). All geographic groups contributed to the increase of DTI reconstruction and to the reduction of expander reconstruction. This was connected to the uncertainties about the future: expander removal could not be scheduled in an emergency setting. Breast-delayed reconstructive procedures, indeed, decreased in all geographic groups, with an overall reduction of 10%, and all the surgeons involved in the study declared that they tried to perform a definitive reconstruction whenever possible.

Our data show a radical change in the surgical management of mastectomy candidates with the implementation of DTI over expander reconstruction. DTI reconstruction was almost mandatory during the pandemic because of the reduction of elective surgeries and the indefinite suspension of delayed reconstructive procedures. Recently, DTI is the preferred approach to breast reconstruction, whenever possible. Casella in 2016 demonstrated that DTI were 35% of all reconstructions after prophylactic nipple-sparing mastectomies (NSM) [11]. This reconstruction presents multiple advantages not only for the surgeons but also for the patients, who are not forced anymore into a long reconstructive path with psychological implications, that should not be underestimated. A DTI reconstruction obviously reduces the need for additional surgeries over time; the reduction of second-stage procedures has also an impact in the healthcare costs and in the availability time for other surgeries and, therefore, for other patients.

Another aspect of breast surgery that was implemented during the pandemic and is becoming part of our clinical practice is the use of local and loco-regional anesthesia with sedation for BCS [11]: during the pandemic it was implemented to reduce the patients’ hospitalization and now is still used, as it reduces time and costs, with greater efficiency in patient management.

The study’s limitations, compared to other studies on the impact of the COVID-19 pandemic on surgery [12], is to consider the number of procedures performed in the entire 2020: in each center the number of procedures has certainly decreased significantly in the first months of 2020 with the first wave of the pandemic and increased afterward to recover, with considerable overload for all the involved surgical staff. All breast care workers lived for months in a high-pressure environment: in the first part of 2020 in most of the hospitals where elective procedures were cancelled the surgical personnel was employed in COVID-19 wards, on a regular or on a volunteer basis [13], experiencing distress both for the lack of protection and for the inability to do their highly specialized work and the request to work out of their comfort zone; later during the year, when elective procedures were allowed, the number of scheduled procedures was increased to recuperate the waiting lists.

## Conclusion

The outburst of the COVID-19 pandemic in 2020 had an impact on breast surgical procedures both due to delays in the diagnostic process and to the uncertainties about the possibility to perform non-urgent surgical procedures such as delayed reconstructions, resulting in an implemented number of mastectomies compared to BCS, an implemented number of immediate breast reconstructions, mainly DTI, and a reduction of expander reconstruction.

## Data Availability

The datasets generated during and/or analysed during the current study are available from the corresponding author on reasonable request.

## References

[1] Chen M, Yuan Y, Zhou Y, Deng Z, Zhao J, Feng F, Zou H, Sun C (2021). Safety of SARS-CoV-2 vaccines: a systematic review and meta-analysis of randomized controlled trials. Infect Dis Poverty.

[2] Bosa I, Catelli A, Castelli M, Ciani O (2022). Response to COVID-19: was Italy (un)prepared?. Health Econ Policy Law.

[3] Pediconi F, Galati F, Bernardi D (2020). Breast imaging and cancer diagnosis during the COVID-19 pandemic: recommendations from the Italian College of Breast Radiologists by SIRM. Radiol med.

[4] Vanni G, Materazzo M, Pellicciaro M (2020). Breast cancer and COVID-19: the effect of fear on patients’ decision-making process. In Vivo.

[5] Freer PE (2021). The impact of the COVID-19 pandemic on breast imaging. Radiol Clin North Am.

[6] Tsang-Wright F, Tasoulis MK, Roche N, MacNeill F (2020). Breast cancer surgery after the COVID-19 pandemic. Future Oncol.

[7] https://www.esmo.org/guidelines/breast-cancer/breast-cancer-in-the-covid-19-era. Accessed 11 April 2020

[8] Vicini E, Galimberti V, Naninato P, Vento AR, Ribeiro Fontana SK, Veronesi P (2020). COVID-19: the European institute of oncology as a “hub" centre for breast cancer surgery during the pandemic in Milan (Lombardy region, northern Italy)—a screenshot of the first month. Eur J Surg Oncol.

[9] Clough KB, Kaufman GJ, Nos C, Buccimazza I, Sarfati IM (2010). Improving breast cancer surgery: a classification and quadrant per quadrant atlas for oncoplastic surgery. Ann Surg Oncol.

[10] Casella D, Calabrese C, Orzalesi L, Gaggelli I, Cecconi L, Santi C, Murgo R, Rinaldi S, Regolo L, Amanti C, Roncella M, Serra M, Meneghini G, Bortolini M, Altomare V, Cabula C, Catalano F, Cirilli A, Caruso F, Lazzaretti MG, Meattini I, Livi L, Cataliotti L, Bernini M (2017). Current trends and outcomes of breast reconstruction following nipple-sparing mastectomy: results from a national multicentric registry with 1006 cases over a 6-year period. Breast Cancer.

[11] Moon EJ, Kim SB, Chung JY, Song JY, Yi JW (2017). Pectoral nerve block (Pecs block) with sedation for breast conserving surgery without general anesthesia. Ann Surg Treat Res.

[12] Medas F, Ansaldo GL, Avenia N, Basili G, Boniardi M, Bononi M, Bove A, Carcoforo P, Casaril A, Cavallaro G, Chiofalo MG, Conzo G, De Pasquale L, Del Rio P, Dionigi G, Dobrinja C, Docimo G, Graceffa G, Iacobone M, Innaro N, Lombardi CP, Palestini N, Pedicini F, Perigli G, Pezzolla A, Scerrino G, Spiezia S, Testini M, Calò PG, SIUEC Collaborative Group (2021). The THYCOVIT (Thyroid Surgery during COVID-19 pandemic in Italy) study: results from a nationwide, multicentric, case-controlled study. Updates Surg..

[13] Li Y, Scherer N, Felix L, Kuper H (2021). Prevalence of depression, anxiety and post-traumatic stress disorder in health care workers during the COVID-19 pandemic: a systematic review and meta-analysis. PLoS ONE.

